# Effects of Shenmai injection on the values of CO, SV, and EF in patients undergoing off-pump coronary artery bypass graft: A randomized, clinical trial

**DOI:** 10.1097/MD.0000000000010085

**Published:** 2018-03-09

**Authors:** Qingxue Liu, Haiyan Wu, Jianjuan Wang, Xi-ming Li

**Affiliations:** a Qingdao University, Qingdao; b Department of Anesthesiology, Linyi City People's Hospital; c Department of Anesthesiology, Shandong Lunan Ophthalmologic Hospital, Linyi, China.

**Keywords:** cardiac output, ejection fraction, off-pump coronary artery bypass graft, Shenmai injection, stroke volume

## Abstract

**Background:**

To explore the effects of Shenmai (SM) injection on the values of cardiac output (CO), stroke volume (SV), and the ejection fraction (EF) in patients treated with off-pump coronary artery bypass graft (OPCABG).

**Methods:**

Forty patients undergoing OPCABG were randomly divided into SM group (n = 20) and the 5% glucose (G) group (n = 20). The control liquids were injected from the beginning of the operation to the start of coronary artery bypass graft (CABG). The values of CO, SV, and EF before induction (t_1_), at the beginning of operation (t_2_), 30 minutes after the start of operation (t_3_), at the beginning of coronary artery bypass graft (t_4_), at the end of coronary artery bypass graft (CABG) (t_5_), and at the end of operation (t_6_) were recorded.

**Results:**

The values of CO, SV, and EF in the patients of SM group at t_3_ to t_6_ were found to be significantly higher than those at t_1_ (*P* < .05). The values of CO, SV, and EF in the patients of G group were found to be increased at t_5_ and t_6_ (*P* < .05). At t_3_ and t_4_, the values of CO, SV, and EF in SM group were significantly higher than those in the G group (*P* < .05).

**Conclusion:**

In patients with OPCABG, the infusion of SM injection can effectively increase the values of CO, SV, and EF and increase the safety of anesthesia management.

## 1 Introduction

Coronary artery bypass grafting (CABG) is the preferred treatment method of coronary artery disease.^[1]^ Off-pump coronary artery bypass graft (OPCABG) can avoid the physiological disturbance with few complications, quick recover, and low cost.^[2,3]^ The study on OPCABG has become a hot spot for surgical treatment of coronary heart disease.^[4,5]^ With the development of rapid postoperative rehabilitation, the great challenge for anesthesiologists and surgeons is to improve heart functions and prevent myocardial ischemia and hypoxia. According to the Traditional Chinese Medicine theory, Shenmai (SM) injection has the function of invigorating *qi*, nourishing *yin* and replenishing bodily fluids.^[6]^ “Shen” and “Mai” are the Chinese abbreviations of red ginseng and ophiopogon japonicas, and a large number of ginseng total saponins are contained in red ginseng. SM injection which contains 2 herbs, namely ginseng and ophiopogon japonicas, is developed and manufactured by Hangzhou Chiatai Qingchunbao Pharnaceutical Co, LTD in China.^[7]^ It is widely used to clinical and the toxicity of SM injection has been evaluated, which is generally considered to be safe to use.^[7,8]^ A study reports that SM has the effects of improving heart functions, balancing blood pressure, dilating the coronary arteries, increasing blood supply, reducing oxygen consumption, eliminating surplus free radicals, and so on.^[9]^ The purpose of this study is to explore the effects of SM injection on the values of cardiac output (CO), stroke volume (SV), and the ejection fraction (EF) in patients treated with OPCABG as well as the safety of anesthesia application. In this study, we concluded that the infusion of SM injection can effectively increase the values of EF, SV, and CO in patients treated with OPCABG and increase the safety of anesthesia management.

## 2 Materials and methods

### 2.1 Materials

A total of 40 patients (age, 46–74 years) scheduled for surgery under general anesthesia were enrolled in this study. Patients with an American Society Anesthesiologists physical status of III or IV, those with a history of adverse effects caused by the study drugs, and those with severe respiratory failure, severe left ventricle dysfunction (EF < 35%), sever uncontrolled hypertension (HTN) or hypotension, pacemaker or intracardiac devices, and bradycardia or arrhythmia were excluded. Patients with bleeding >1000 mL during or after surgery, patients with >4 grafts were also excluded. This study was approved by the Medical Ethics Committee of Linyi City People's Hospital (NO: KY2015004), Linyi, China.

### 2.2 Methods

After obtaining informed consent from all the patients, we randomized them into 2 groups. The SM group was given 60 mL of SM injection which was diluted to 150 mL with 5% glucose (G) solution when the skin was cut at the speed of 150 mL/hour, while the G group was given 150 mL of 5% G at the same speed and the same time as the SM group. Patients of 2 groups were continuously given dopamine and nitroglycerin. After successful artery puncture under local anesthesia, the Vigileo monitor was connected to the patients via a FloTrac pressure sensor, and the data were collected continuously after calibration.

General anesthesia was induced and maintained by the same method. After establishing full cardiovascular monitoring, general anesthesia was induced with sufentanyl 1 μg/kg, midazolam 0.05 mg/kg, lidocaine, and etomidate 1 to 2 mg/kg until loss of eyelid reflex. Orotracheal intubation was facilitated by 0.1 mg/kg cisatracurium. Routine airway and ventilator management were used as appropriate for the type of surgery. Anesthesia was maintained with continuous infusion of remifentanyl 0.2 μg/kg hour, propofol 4 mg/kg hour, and sevoflurance 1% to 2% until patient transfers to open heart ICU. After the mechanical ventilation, transesophageal echocardiography was used to guide the puncture of right internal jugular vein. BIS monitoring and nasopharyngeal temperature and urine monitoring were given. A total of 5 μg/kg min of dopamine, 0.6 μg/kg min of nitroglycerin, and 0.1 μg/kg min of phenylephrine were chosen as vasoactive drug.

### 2.3 Monitoring indicators

The values of CO, SV, and EF of the 2 groups were recorded before the induction of anesthesia (t_1_), at the start of surgery (t_2_), 30 minutes after the start of surgery (t_3_), at the beginning of coronary artery bypass graft (t_4_), at the end of coronary artery bypass graft (t_5_), and at the end of surgery (t_6_).

### 2.4 Statistical analysis

All the statistical analyses were using the SPSS (version 20, IBM logo,ibm.com). All data are presented as mean ± standard deviation. Demographic data were analyzed by Student *t* test or Mann–Whitney *U* test. Analysis of variance for repeated measures was used to analyze hemodynamic changes and CO, SV, EF, over time between 2 groups.

## 3 Results

### 3.1 Compared to G injection, SM injection was more effective on the values of CO, SV, and EF

**Figure 1:**
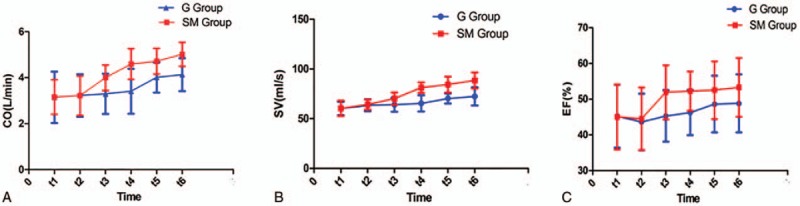
The values of CO, SV, and EF. (A) The values of CO in the SM group and the G group at t_1_, t_2_, t_3_, t_4_, t_5_, and t_6_. (B) The values of SV in the SM group and the G group at t_1_, t_2_, t_3_, t_4_, t_5_, and t_6_. (C) The values of EF in the SM group and the G group at t_1_, t_2_, t_3_, t_4_, t_5_, and t_6_. CO = cardiac output, EF = ejection fraction, G = glucose, SM = Shenmai, SV = stroke volume.

We firstly compared the general information of the 2 groups before surgery, resulting that general information of the 2 groups showed no significant differences (Table 1). The variables of coronary artery disease and EF in patients of the study were not significantly different in 2 groups of study (*P* > .05) (Table 2). All the patients were treated with the same group of cardiac surgeons, and the surgical methods were all the same. Effects of hemodynamics and CO, SV, EF in the SM group were detected, and the results showed that the values of CO, SV, and EF in the SM group at t_3_ to t_6_ were found to be significantly higher than those at t_1_ (*P* < .05) (Table 3 and Fig. 1). Equally, effects of hemodynamics and CO, SV, EF in the G group were detected, and the results showed that the values of CO, SV, and EF in the G group were found to be increased at t_5_ and t_6_ (*P* < .05) (Table 3 and Fig. 1). However, the values of CO, SV, and EF in the SM group were significantly higher than those in the G group (*P* < .05) at t_3_ and t_4_ (Table 3 and Fig. 1), indicating that compared to G injection, SM injection was more effective on the values of CO, SV, and EF.

**Table 1:**
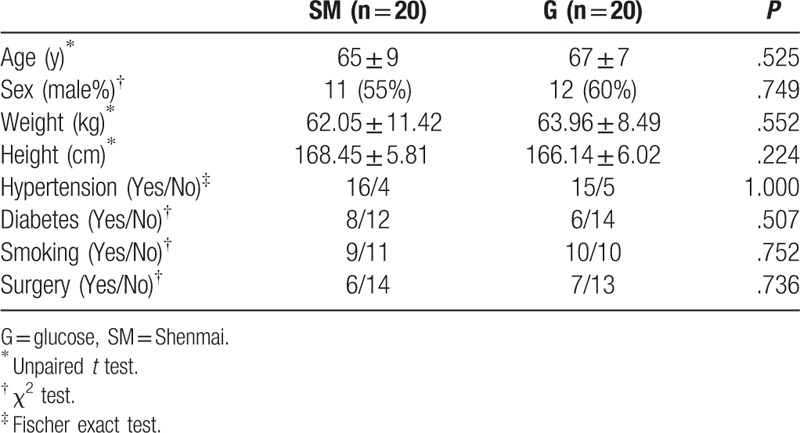
Comparison of general information.

**Table 2:**
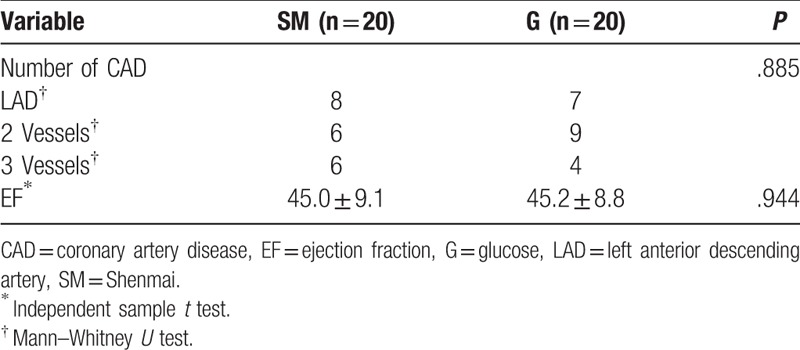
Variables of coronary artery disease and ejection fraction.

**Table 3:**
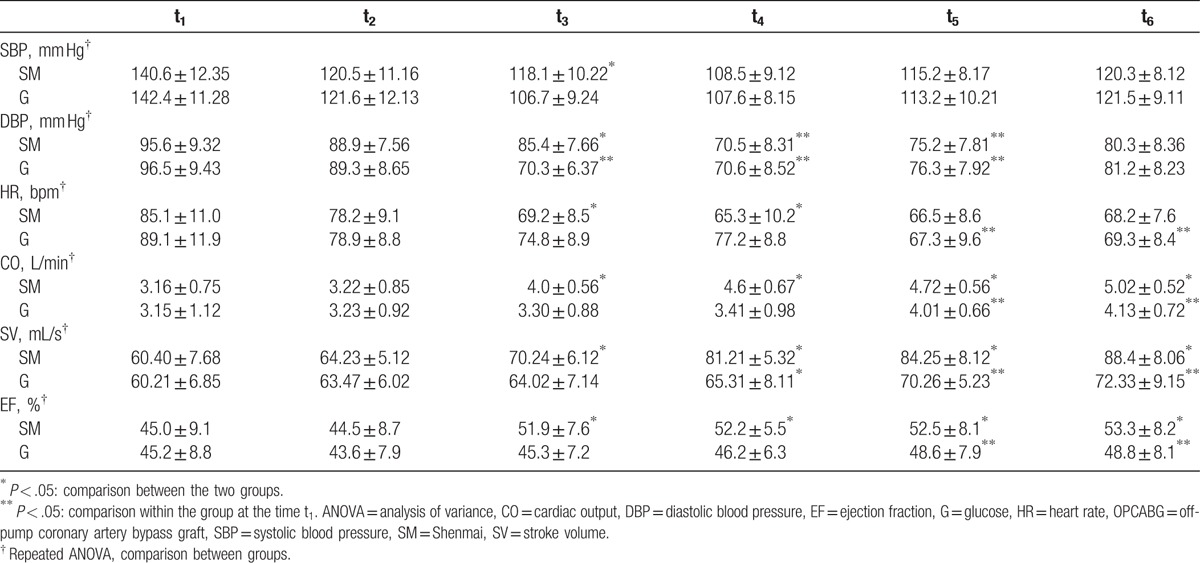
Effects of hemodynamics and CO, SV, and EF 
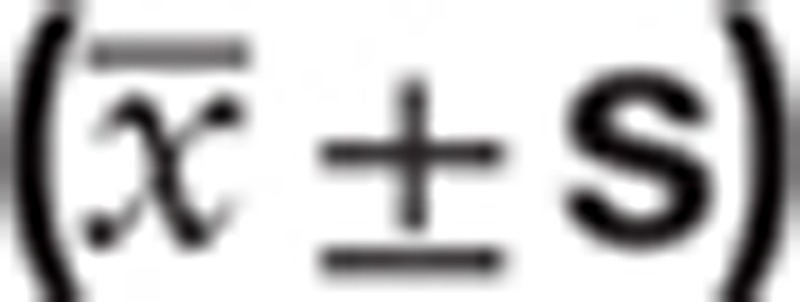
.

## 4 Discussion

SM injection stemmed from ancient traditional Chinese prescription of *shengmaisan*. It contains ginseng saponin, ginseng polysaccharides, ophiopogonin, organic acids, and so on.^[6]^ The studies have confirmed that it can promote coronary blood flow, reduce myocardial oxygen consumption,^[10]^ inhibit apoptosis of myocardial hypoxia and provide myocardial protection,^[11–13]^ reduce stress, improve antioxidant capacity, regulate, and promote immune functions.^[14,15]^ Cardiac effect of SM injection is stable and long-lasting, which is consistent with the perioperative medication characteristics of coronary artery bypass surgery. Tian et al^[16]^ has confirmed that SM injection given 1 week before surgery and 1 week after CABG can improve cardiac functions and protect the heart.

Systemic inflammatory response may be caused by surgical trauma, blood contacting foreign body on the surface of cardiopulmonary bypass devices, and ischemia-reperfusion injury. After surgery, the clinical manifestations were fever, leukocytosis, increased capillary permeability, leading to serious hyperdynamic circulation, multiple organ dysfunction, postoperative complications, and high mortality.^[17]^ However, OPCABG has the advantages of low probability of postoperative complications, early extubation, and quick recovery, which needs strict demand for proper anesthesia management. Perioperative reasonable application of the cardiovascular active drug and maintaining stable hemodynamics are the keys to successful OPCABG.^[18]^

Currently, research on the application of SM injection in nonpump coronary artery bypass surgery has not yet been reported. In this study, both groups received the same surgical procedure and anesthesia, and SM injection was given and the cardiac function was observed at the very beginning of surgical incision. The results showed that in SM group, the value of CO increased by 20% in 60 minutes (at t_2_–t_4_), the value of SV increased by 10%, the value of EF increased by 10%, but the value of heart rate decreased, the myocardial oxygen consumption also decreased significantly (*P* < .05). SM injection improved the left ventricular filling and left ventricular compliance significantly, promoted the heart functions, maintained the hemodynamic stability, and made the management safer.

Studies have confirmed that SM injection can inhibit myocardial ischemia, reduce myocardial oxygen consumption and myocardial apoptosis, and protect heart function.^[19,20]^ In addition, it can also enhance diastolic function and improve heart functions after heart valve replacement.^[21,22]^ It has unique effect on patients with heart failure.^[23]^ Intraoperative infusion of SM injection has good security without obvious adverse effects.^[24]^

In summary, patients receiving coronary artery bypass surgery have poor cardiac functions. Under surgical and anesthetic stress, cardiac functions are significantly inhibited, which need drugs to enhance the tolerance of surgery and anesthesia. SM injection can improve the values of CO, SV, and EF in patients during OPCABG, which are of great significance in maintaining hemodynamic stability and prognosis of myocardial protection. The molecular mechanisms of SM injection in improving cardiac functions in patients treated with OPCABG remains to be further studied.
